# Pharmacotherapy of 1,044 inpatients with posttraumatic stress disorder: current status and trends in German-speaking countries

**DOI:** 10.1007/s00406-020-01223-x

**Published:** 2020-12-28

**Authors:** Matthias A. Reinhard, Johanna Seifert, Timo Greiner, Sermin Toto, Stefan Bleich, Renate Grohmann

**Affiliations:** 1 Department of Psychiatry and Psychotherapy, LMU University Hospital Munich, Nußbaumstr. 7, 80336 Munich, Bavaria Germany; 2 Department of Psychiatry, Social Psychiatry and Psychotherapy, Carl-Neuberg-Straße 1, 30625 Hannover, Germany; 3 Brandenburg Medical School, Immanuel Klinik Rüdersdorf, University Clinic for Psychiatry and Psychotherapy, Seebad 82/83, 15562 Rüdersdorf bei Berlin, Germany

**Keywords:** Posttraumatic stress disorder, PTSD, Pharmacotherapy, AMSP, Guideline

## Abstract

Posttraumatic stress disorder (PTSD) is a debilitating psychiatric disorder with limited approved pharmacological treatment options and high symptom burden. Therefore, real-life prescription patterns may differ from guideline recommendations, especially in psychiatric inpatient settings. The European Drug Safety Program in Psychiatry (“Arzneimittelsicherheit in der Psychiatrie”, AMSP) collects inpatients’ prescription rates cross-sectionally twice a year in German-speaking psychiatric hospitals. For this study, the AMSP database was screened for psychiatric inpatients with a primary diagnosis of PTSD between 2001 and 2017. *N* = 1,044 patients with a primary diagnosis of PTSD were identified with 89.9% taking psychotropics. The average prescription rate was 2.4 (standard deviation: 1.5) psychotropics per patient with high rates of antidepressant drugs (72.0%), antipsychotics drugs (58.4%) and tranquilizing drugs (29.3%). The presence of psychiatric comorbidities was associated with higher rates of psychotropic drug use. The most often prescribed substances were quetiapine (24.1% of all patients), lorazepam (18.1%) and mirtazapine (15.0%). The use of drugs approved for PTSD was low (sertraline 11.1%; paroxetine 3.7%). Prescription rates of second-generation antipsychotic drugs increased, while the use of tranquilizing drugs declined over the years. High prescription rates and extensive use of sedative medication suggest a symptom-driven prescription (e.g., hyperarousal, insomnia) that can only be explained to a minor extent by existing comorbidities. The observed discrepancy with existing guidelines underlines the need for effective pharmacological and psychological treatment options in psychiatric inpatient settings.

## Introduction

Posttraumatic stress disorder (PTSD) is a psychiatric disorder that develops following exposure to a traumatic event and is a prevalent reason for inpatient psychiatric admission. According to the International Classification of Diseases (ICD-10) [1], trauma is defined as a stressful event or situation of exceptionally threatening or catastrophic nature. In addition, diagnostic criteria of PTSD include (1) re-experiencing the traumatic event as intrusive “flashbacks” or vivid memories, (2) avoidance of trauma-related circumstances and (3) increased arousal (“hyperarousal”) or psychological sensitivity [1]. Besides these symptoms, PTSD is associated with sleep disturbance due to insomnia and nightmares [2], anxious and depressed affect, negative cognitions and rumination. As a consequence, comorbid psychiatric disorders are common in patients with PTSD (e.g., depression, anxiety and substance use disorders [3]).

PTSD is a prevalent psychiatric disorder in Germany with an estimated 1-month prevalence of 1.3–3.4% [4] and a lifetime prevalence of 2.9% [5]. Lifetime prevalence of PTSD worldwide ranges between 1.3 and 8.8% [6]. Lifetime exposure to a traumatic event seems low in Germany (23.8% according to [5]) compared to 70.4% of humans described in the WHO World Mental health sample of 24 countries (sample includes Germany [7]). Despite exposure to trauma, most people do not develop PTSD following a traumatic event [6]. The risk seems to depend on the type of trauma with highest risk for developing PTSD after experiencing interpersonal violence trauma [7]. Frequency of exposure to different types of traumatic events differs between men and women with higher prevalence and risk of developing PTSD in women [3, 6, 7]. Symptoms of PTSD may be transient and remit within several weeks or months after the traumatic event. However, up to one third of PTSD patients has persisting symptoms over 6 years [3].

A variety of psychological and pharmacological treatment options are available for PTSD. Currently, trauma-focused psychotherapy is recommended as first-line treatment for PTSD as it outperforms both non-trauma-focused psychotherapy and medication [8]. However, this result has been challenged by a recent study with *n* = 223 participants that found no difference between prolonged exposure therapy, sertraline with optimized medication enhancement or the combination of prolonged exposure therapy with sertraline [9]. Psychotropic medication is widely used to support treatment, as trauma-focused psychotherapy is often not sufficient on its own, especially in the beginning, not available on the short term or not tolerated [10]. Within a large cohort of American Veterans with PTSD observed between 2004 and 2013, each veteran was prescribed an average of 3.5 psychotropic drugs [11]. The most commonly prescribed drug classes in this cohort were antidepressant drugs (81.0%) followed by sedative hypnotic drugs including benzodiazepines (38.9%), opioids (36.9%), antiepileptic drugs (24.9%) and atypical antipsychotic drugs (21.8%). The most common antidepressant substances in 2013 in this cohort were sertraline (31.2%) and trazodone (29.7% [11]).

Regarding the evidence of pharmacological treatment, two meta-analyses found that the use of psychotropic drugs was superior to placebo in the treatment of PTSD [10, 12]. Whereas fluoxetine, paroxetine and venlafaxine showed a small effect compared to placebo regarding clinical symptoms, Hoskins et al. [10] found no clear evidence for bromaforine, olanzapine, sertraline and topiramate. Using the response rate as outcome variable, Gu et al. [12] found an effect for fluoxetine, paroxetine and sertraline. Finally, Lee et al. [8] describe a small effect for sertraline, venlafaxine and nefazodone. There is only insufficient evidence supporting the use of most other substances [10], e.g., prazosin/doxazosin for treating insomnia in PTSD [13, 14]. The use of benzodiazepines is even relatively contraindicated despite their short-term benefits because of adverse effects (mainly addictive potential and rebound symptoms) and may lead to a lower success rate of psychotherapy [15].

According to the above-mentioned findings, the German guidelines of the “Arbeitsgemeinschaft der Wissenschaftlichen Medizinischen Fachgesellschaften e.V.” (AWMF [16]) recommend trauma-focused psychotherapy as treatment of choice. Pharmacotherapy, not as first-line or sole treatment, should consist of sertraline, paroxetine or venlafaxine. However, in Germany only sertraline and paroxetine are approved for the treatment of PTSD. Venlafaxine is recommended in case of comorbid depression or anxiety disorder, otherwise its “off-label” use has to be stated.

Other existing guidelines such as the NICE guidelines (http://www.nice.org.uk/guidance/ng116) similarly recommend selective serotonin reuptake inhibitors (SSRI: sertraline, fluoxetine and paroxetine) or venlafaxine, however not as first-line treatment. Antipsychotic drugs (such as risperidone) are seen as potentially useful for the management of PTSD symptoms but should only be an addition to psychotherapy.

Despite existing guidelines, discrepancies between the recommendations and real-life pharmacological treatment of PTSD prevail [17]. This may especially be the case within a psychiatric inpatient setting in which patients with particularly high symptom burden, more comorbidities or preceding failure of outpatient treatment are treated. Existing data is mainly limited to reports of American US veteran outpatients. Unfortunately, prescription data among patients without combat-induced trauma and outside the US is currently hardly available. Therefore, the current study aimed to assess the actual clinical prescription practice for inpatients with PTSD for a broader and more diverse patient collective better reflecting real-life conditions in German-speaking countries. In addition, changes in prescription patterns over time were analyzed in order to detect trends that may point toward increased awareness of clinical guidelines or effectiveness of specific substances.

## Materials and methods

### Data source

Data for this study was provided by the European Drug Safety Program in Psychiatry (“Arzneimittelsicherheit in der Psychiatrie”, AMSP). AMSP is a multicenter drug surveillance program that collects data on (1) drug prescription and (2) severe adverse drug reactions in the natural setting of routine psychiatric inpatient treatment (for a detailed description, see [18, 19]). In brief, AMSP was designed in 1993 to improve drug safety within the psychiatric inpatient setting and to gain insight into the utilization patterns of psychotropic drug utilization in psychiatric hospitals. AMSP does not interfere with the ongoing treatment. Since 1993, the number of participating hospitals that provides data has increased. In 2017, 52 psychiatric institutions in Germany, Austria and Switzerland contributed to AMSP. Twice a year on given reference days, participating institutions record drug prescriptions for all psychiatric inpatients currently in treatment. The collected data contains all prescribed psychotropic and non-psychotropic medication (daily dosage) as well as sex, age and primary psychiatric diagnosis according to the ICD-10. The primary diagnosis is the main reason for admission to inpatient treatment. Since 2007, secondary and tertiary diagnoses have been additionally assessed. Diagnoses are coded by the treating physician under supervision of an experienced senior psychiatrist. Data is then transferred to the anonymized AMSP data bank. Data evaluation and analysis of the AMSP database have been approved by both the Ethics Committee of the University of Munich and the Ethics Committee of the Hannover Medical School (#8100_BO_S_2018).

### Study population

All inpatients with a primary diagnosis of PTSD (ICD-10: F43.1) between 2001 and 2017 were selected from the AMSP database. Because PTSD is difficult to diagnose and potentially overseen especially when it is not the main reason for hospitalization, analysis was limited to patients with a primary diagnosis. 2001 was chosen to guarantee a consistent definition of PTSD criteria due to the implementation of ICD-10 and a change of diagnostic criteria compared to ICD-9. Data stems from a total of 94 psychiatric hospitals.

Psychotropic medication was classified as following: antidepressant drugs, antipsychotic drugs, tranquilizing drugs (mostly benzodiazepines), hypnotic drugs (mostly benzodiazepine-like drugs, e.g., zopiclone, zolpidem, and the benzodiazepines nitrazepam, flurazepam and lormetazepam), antiepileptic drugs, lithium, antiparkinsonian medication (mostly biperiden), nootropics and other psychotropic drugs (e.g., methylphenidate). Antidepressant drugs were further divided into SSRI, selective serotonin noradrenaline reuptake inhibitors (SSNRI), noradrenergic and specific serotonergic antidepressants (NaSSA) and tricyclic antidepressants (TCA). Antipsychotic drugs were grouped into low-potency first-generation antipsychotics (FGA), high-potency FGA and second-generation antipsychotics (SGA). Furthermore, the prescription rate of single drugs is reported for all drugs used in > 5% of all patients with PTSD and additionally also for paroxetine as one of the approved drugs in Germany, Austria and Switzerland.

### Data analysis

Data was analyzed with Excel 2019 and SPSS 25.0 (https://www.ibm.com/de-de/products/spss-statistics). There was no missing data. Descriptive data is presented as the number of patients taking a specific substance or at least one substance within a psychotropic drug group. Percentages refer to the whole sample (including patients without medication) if not otherwise specified. The effects of sex and comorbidity were analyzed with Chi-squared tests for two groups or *t* tests for independent groups as appropriate. To analyze the effect of age on prescription rates, we performed separate logistic regression analyses with age as predictor and psychotropic drug groups as dependent variable. Finally, we analyzed time trends and performed logistic regression analyses with year of prescription as predictor to model prescription rates of psychotropic drug groups. As the primary focus of this study is on description and modeling, tests of significance were used solely in a descriptive way.

## Results

### Sample

A total of 1,044 inpatients with a primary diagnosis of PTSD were identified in the AMSP database, which contains data from a total 147,481 patients. Therefore, PTSD patients comprised 0.7% of all patients. Data stems from 63 psychiatric hospitals in Germany, 13 in Austria and 18 in Switzerland.

The sample consisted of 329 males (31.5%) and 715 females (68.5%). Mean age was 37.2 ± 12.8 years with a minimum age of 15 and maximum of 94 years. Seven patients were younger than 18 years. There was no significant mean age difference between men and women [*t*(1042) = 1.57, *p* = 0.12]. The majority of patients was between 21 and 50 years old (74.5%) with 26.7% between 21 and 30 years, 24.6% between 31 to 40 years and 23.2% between 41 and 50 years.

Comorbid psychiatric diagnoses were available as of 2007 (772 patients with a primary diagnosis of PTSD since 2007) with 58.4% of monitored patients suffering from a psychiatric comorbidity. Most common psychiatric comorbidities alongside a primary diagnosis of PTSD were affective disorders (ICD-10 F3: 27.8%; including 10.8% with major depressive episode, 16.6% with recurrent depressive disorder, and 0.4% with bipolar affective disorder) followed by personality disorders (ICD-10 F6: 14.2%; including borderline personality disorder, BPD: 8.2%) and psychoactive substance abuse (ICD-10 F1: 13.6%; including 6.3% with an alcohol related disorder; 3.2% sedatives; 2.2% cannabis; 1.3% opioid related). Further, 11.3% of patients suffered from a comorbid F4 diagnosis (anxiety disorder: 3.4%; dissociative disorder: 4.5%; somatoform disorder: 3.1%). A small number of patients had comorbid behavioral syndromes (ICD-10 F5: 3.1%, e.g., eating disorder) or a diagnosis of schizophrenia/delusional disorder (ICD-10 F2: 2.3%). Women more frequently suffered from a psychiatric comorbidity than men [61.2% vs. 53.0%, *χ*^2^(1, *n* = 772) = 4.55, *p* = 0.03].

### Prescription rates

94.0% of all patients received some type of medication (including non-psychotropic drugs) and 89.9% were prescribed at least one psychotropic drug (Table 1). 19.7% received one psychotropic drug, 70.2% received two or more. The average number of prescribed psychotropics was 2.4 ± 1.5 per patient. Most commonly prescribed psychotropics per patient were antidepressant drugs (72.0%) and antipsychotic drugs (58.4%), followed by tranquilizing drugs (29.3%, mainly benzodiazepines), antiepileptic drugs (18.4%) and hypnotic drugs (12.3%). The most often prescribed subgroup of antidepressant drugs per patient was SSRIs (32.5%), followed by SSNRIs (19.4%), NaSSAs (15.2%) and TCAs (14.2%). More patients were prescribed SGAs (44.2%) than low-potency FGAs (22.9%) or high-potency FGAs (5.2%). 19.8% of patients received two or more antidepressant drugs (average number of prescribed antidepressant drugs: 0.9 ± 0.7). 17.0% received more than one antipsychotic drug (average number: 0.8 ± 0.8). Antidepressant drugs were most often combined with antipsychotic drugs (45.2% of patients), tranquilizing drugs (22.8%) or another antidepressant drug (19.8%).

**Table 1:** Percentage of prescribed drug groups for men and women from 2001 to 2017

	Total % (*n* = 1044)	Men % (*n* = 329)	Women % (*n* = 715)	*χ*^2^(1, *n* = *1044)*	*p*
Any medication	94.0% (981)	96.7% (318)	92.7% (663)	6.14	0.01*
Any psychotropics	89.9% (939)	91.8% (302)	89.1% (637)	1.82	0.18
Antidepressant drugs	72.0% (752)	71.7% (236)	72.2% (516)	0.02	0.88
SSRI	32.5% (339)	30.7% (101)	33.3% (238)	0.69	0.41
SSNRI	19.4% (203)	20.4% (67)	19.0% (136)	0.26	0.61
NaSSA	15.2% (159)	19.1% (63)	13.4% (96)	5.72	0.02*
TCA	14.2% (148)	10.6% (35)	15.8% (113)	4.94	0.03*
“Other antidepressants”	11.1% (116)	9.4% (31)	11.9% (85)	1.39	0.24
Antipsychotic drugs	58.4% (610)	59.3% (195)	58.0% (415)	0.14	0.71
SGA	44.2% (461)	45.9% (151)	43.4% (310)	0.59	0.44
Low-potency FGA	22.9% (239)	20.1% (66)	24.2% (173)	2.18	0.14
High-potency FGA	5.2% (54)	5.8% (19)	4.9% (35)	0.36	0.55
Tranquilizing drugs	29.3% (306)	28.6% (94)	29.7% (212)	0.13	0.72
Benzodiazepines	26.9% (281)	26.4% (87)	27.1% (194)	0.05	0.82
Tricyclic tranquilizers	1.3% (15)	1.2% (4)	1.5% (11)	0.17	0.68
Plant-based tranquilizers	0.7% (7)	0.9% (3)	0.6% (4)	0.42	0.52
Hypnotic drugs	12.3% (128)	11.6% (38)	12.6% (90)	0.23	0.64
Benzodiazepine analogues	8.9% (93)	9.1% (30)	8.8% (63)	0.03	0.87
Benzodiazepines	1.9% (20)	1.8% (6)	2.0% (14)	0.02	0.88
Plant-based hypnotics	1.1% (12)	0.9% (3)	1.3% (9)	0.24	0.63
Antiepileptic drugs	18.4% (192)	16.1% (53)	19.4% (139)	1.67	0.20
Lithium	2.4% (25)	0.6% (2)	3.2% (23)	6.56	0.01*
Antiparkinson drugs	2.9% (30)	2.4% (8)	3.1% (22)	0.34	0.56
Nootropics	0.5% (5)	0.9% (3)	0.3% (2)	1.89	0.17
Other psychotropics	1.6% (17)	2.1% (7)	1.4% (10)	0.75	0.39

*SSRI* selective serotonin reuptake inhibitor, *SSNRI* selective serotonin noradrenaline reuptake inhibitors, *NaSSA* noradrenergic and specific serotonergic antidepressants, *TCA* tricyclic antidepressants, Other antidepressants: trazodone *n* = 71, agomelatine *n* = 31, bupropion *n* = 14, reboxetine *n* = 3; *SGA* second-generation antipsychotics, *FGA* first-generation antipsychotics

*p < 0.05, reported *p *values are not corrected for multiple comparisons

The most frequently prescribed psychotropic drugs were quetiapine (24.1% of all patients), lorazepam (18.1%) and mirtazapine (15.0%). Table 2 shows the most commonly prescribed psychotropics and average daily dose of each substance. The rates of the approved drugs sertraline and paroxetine were 11.1% and 3.7%, respectively. Doxazosin/prazosin was prescribed to 15 patients (1.4%).

**Table 2:** Prescription rate of the most frequently prescribed psychotropics (> 5% of all patients) for men and women with mean dosage and standard deviation

Substance	All patients (*N* = 1044)	Mean dosage [mg]
Quetiapine	24.1% (252)	272.4 ± 208.7
Lorazepam	18.1% (189)	2.2 ± 1.8
Mirtazapine	15.0% (157)	30.5 ± 14.9
Venlafaxine	12.7% (133)	185.0 ± 79.7
Sertraline	11.1% (116)	99.1 ± 49.6
Olanzapine	10.0% (104)	11.0 ± 7.1
Escitalopram	8.1% (85)	15.0 ± 6.7
Trazodone	6.8% (71)	149.3 ± 77.9
Citalopram	6.7% (70)	29.8 ± 13.0
Duloxetine	6.3% (66)	85.9 ± 34.6
Risperidone	5.8% (61)	2.5 ± 1.5
Chlorprothixene	5.6% (59)	107.7 ± 151.1
Trimipramine	5.7% (60)	93.6 ± 83.3
Pregabalin	5.2% (54)	270.0 ± 182.5
…	…	…
Paroxetine	3.7% (39)	32.3 ± 15.8

Further included is paroxetine (approved in Germany, Austria and Switzerland)

Men were more likely to be prescribed medication than women when including non-psychotropic medication: 96.7% vs. 92.7% [*χ*^2^(1, *n* = 1044) = 6.14, *p* = 0.01; see Table 1], however, there was no difference regarding the average number of psychotropics [men: 2.3 ± 1.4; women: 2.5 ± 1.6, *t*(1042) = 1.27, *p* = 0.20]. Male patients more often received NaSSAs than women [19.1% vs. 13.4%, *χ*^2^(1, *n* = 1044) = 5.72, *p* = 0.02], while TCAs were more often prescribed to women compared to men [15.8% vs. 10.6%, *χ*^2^(1, *n* = 1044) = 4.94, *p* = 0.03]. In addition, women were more frequently treated with lithium [3.2% vs 0.6%, *χ*^2^(1, *n* = 1044) = 6.56, *p* = 0.01].

Patients’ age also affected prescription rates: Logistic regression analyses revealed that the prescription rate of antidepressant drugs increased with age (*B* = 0.02, SE = 0.01, Wald = 17.87, *p* < 0.001) with a specifically significant increase of SSNRI (*B* = 0.02, SE = 0.01, Wald = 15.00, *p* < 0.001) and TCA (*B* = 0.02, SE = 0.01, Wald = 5.38, *p* = 0.02). Age did not affect prescription rates of SSRI, NaSSA and “other antidepressant drugs”. In addition, prescription of antiepileptic drugs increased with age (*B* = 0.01, SE = 0.01, Wald = 4.14, *p* = 0.04). In contrast, antipsychotic, tranquilizing and hypnotic drugs showed neither an increase nor decrease with age. To illustrate these findings, Table 3 shows the number of patients per age group treated with a substance within the most often prescribed drug groups.

**Table 3:** Rates of prescribed psychotropic drug groups per patient for different age groups

Age group	14–30 (*n* = 371)	31–40 (*n* = 257)	41–50 (*n* = 242)	51–95 (*n* = 174)
Psychotropics	87.9% (326)	88.3% (227)	93.0% (225)	92.5% (161)
Antidepressant drugs	65.0% (241)	73.5% (189)	75.2% (182)	80.5% (140)
Antipsychotic drugs	58.5% (217)	56.4% (145)	66.1% (160)	50.6% (88)
Antiepileptic drugs	15.1% (56)	16.7% (43)	24.8% (60)	19.0% (33)
Tranquilizing drugs	27.0% (100)	31.1% (80)	33.9% (82)	25.3% (44)
Hypnotic drugs	12.1% (45)	11.7% (30)	14.5% (35)	10.3% (18)

Furthermore, we analyzed whether the presence of psychiatric comorbidity was related to prescription rates (see Table 4). In general, patients with additional psychiatric diagnoses received psychotropic drugs more often than patients without psychiatric comorbidity [92.2% vs. 86.0%, *χ*^2^(1, *n* = 772) = 7.91, *p* = 0.005]. By trend, patients with a psychiatric comorbidity were more likely to be prescribed SSRI [33.0% vs. 27.1%, *χ*^2^(1, *n* = 772) = 3.11, *p* = 0.08] than patients without additional psychiatric diagnoses. Substances classified as “other antidepressant drugs” (mainly trazodone, reboxetine and agomelatine) were more often prescribed to patients with psychiatric comorbidity [16.2% vs. 10.6%, *χ*^2^(1, *n* = 772) = 4.92, *p* = 0.03]. Finally, patients with comorbid psychiatric diagnosis had higher prescription rates of antipsychotic drugs [65.0% vs. 57.9%, *χ*^2^(1, *n* = 772) = 3.93, *p* = 0.04] and lithium [4.2% vs. 1.2%, *χ*^2^(1, *n* = 772) = 5.71, *p* = 0.02].

**Table 4:** Prescription rates for patients without or with comorbid psychiatric diagnosis between 2007 and 2017

	All patients (*n* = 772)	Without additional diagnosis (*n* = 321)	With additional diagnosis (*n* = 451)	*χ*^2^ (1,* n* = 772)	*p*
Any medication	94.4% (724)	92.5% (297)	94.7% (427)	1.49	0.22
Any psychotropics	89.6% (692)	86.0% (276)	92.2% (416)	7.91	0.005**
Antidepressant drugs	71.2% (550)	68.6% (220)	73.2% (330)	1.97	0.16
SSRI	30.6% (236)	27.1% (87)	33.0% (149)	3.11	0.08
SSNRI	22.8% (176)	22.1% (71)	23.3% (105)	0.14	0.70
NaSSA	15.0% (116)	17.8% (57)	13.1% (59)	3.21	0.07
TCA	12.0% (93)	9.3% (30)	14.0% (63)	3.78	0.05
“Other antidepressants”	13.9% (107)	10.6% (34)	16.2% (73)	4.92	0.03*
Antipsychotic drugs	62.0% (479)	57.9% (186)	65.0% (293)	3.93	0.04*
SGA	49.0% (378)	46.7% (150)	50.6% (228)	1.10	0.30
Low-potency FGA	22.5% (174)	20.2% (65)	24.2% (109)	1.65	0.20
High-potency FGA	4.4% (34)	3.7% (12)	4.9% (22)	0.58	0.45
Tranquilizing drugs	28.1% (217)	27.1% (87)	28.8% (130)	0.28	0.60
Hypnotic drugs	12.2% (94)	10.9% (35)	13.1% (59)	0.83	0.36
Antiepileptic drugs	19.3% (149)	16.2% (52)	21.5% (97)	3.39	0.07
Lithium	3.0% (23)	1.2% (4)	4.2% (19)	5.71	0.02*

*SSRI* selective serotonin reuptake inhibitor, *SSNRI* selective serotonin noradrenaline reuptake inhibitors, *NaSSA* noradrenergic and specific serotonergic antidepressants, *TCA* tricyclic antidepressants, *SGA* second-generation antipsychotics, *FGA* first-generation antipsychotics

*p < 0.05,**p < 0.01, reported *p *values are not corrected for multiple comparisons

As 86.0% of patients without comorbidity had psychotropic medication, we further analyzed this subgroup (*n* = 321). Similar to the total sample, this subgroup was most often treated with antidepressant drugs (68.6%) and antipsychotic drugs (57.9%), followed by tranquilizing drugs (27.1%), antiepileptic drugs (16.2%) and hypnotic drugs (10.9%; see Table 3). Again, the most frequently prescribed drugs were quetiapine (20.4% of this subgroup), lorazepam (12.8%) and mirtazapine (12.4%). Only 2.2% of patients took sertraline and 0.0% paroxetine. Thus, 83.8% of these patients had psychotropics that were not approved for the treatment of PTSD. Regarding sex differences, women were more likely to receive hypnotic drugs than men in this subgroup [*χ*^2^(1, *n* = 451) = 4.82, *p* = 0.03]. Finally, logistic regression analyses revealed that prescription rates of antidepressant drugs increased with age (*B* = 0.02, SE = 0.01, Wald = 4.43, *p* = 0.04), especially SSRIs (*B* = 0.02, SE = 0.01, Wald = 4.27, *p* = 0.04). Age did not affect use of antipsychotic, tranquilizing, antiepileptic and hypnotic drugs.

### Time trends

Logistic regression analysis revealed that the overall prescription rate of antidepressant drugs per patient remained consistent over time (*B* = − 0.01, SE = 0.02, Wald = 0.20, *p* = 0.66). However, a significant decrease was especially seen for prescription rates of TCAs (*B* = − 0.07, SE = 0.02, Wald = 13.47, *p* < 0.001). Simultaneously, the prescription of SSNRIs and “other antidepressant drugs” significantly increased over time (SSNRI: *B* = 0.08, SE = 0.02, Wald = 18.40, *p* < 0.001; Other: *B* = 0.14, SE = 0.02, Wald = 31.22, *p* < 0.001, mainly trazodone and agomelatine).The overall prescription rate of antipsychotic drugs per patient increased over time (*B* = 0.05, SE = 0.01, Wald = 12.41, *p* < 0.001) with a significant increase of the prescription of SGAs (*B* = 0.08, SE = 0.01, Wald = 35.21, *p* < 0.001) and a decline of prescription rates of high potency FGAs (*B* = − 0.03, SE = 0.02, Wald = 4.31, *p* = 0.04). The prescription rate of tranquilizing drugs as a group showed a decline (*B* = − 0.04, SE = 0.01, Wald = 7.92, *p* = 0.005). Hypnotic and antiepileptic drugs were consistently prescribed over time.

**Fig. 1:**
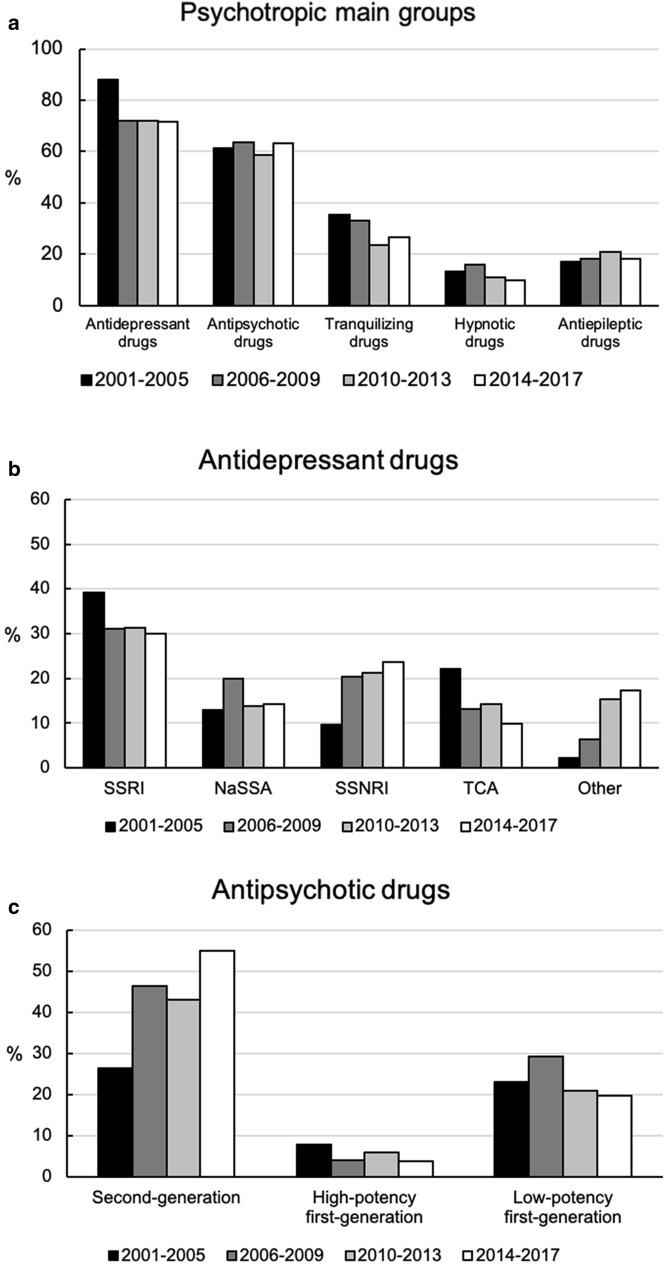
Time trends for prescription rates of **a** main groups of psychotropic drugs, **b** antidepressant drugs: selective serotonin reuptake inhibitors (SSRI), noradrenergic and specific serotonin reuptake inhibitors (NaSSA), selective serotonin noradrenaline reuptake inhibitors (SSNRI), tricyclic antidepressants (TCA) and “other antidepressants” and **c** antipsychotic drugs

To illustrate time trends, four time periods were created (2001–2005: *n* = 217; 2006–2009: *n* = 250; 2010–2013: *n* = 240; 2014–2017: *n* = 337). Figure 1 shows the prescription rates of psychotropics (Fig. 1a), subgroups of antidepressant drugs (Fig. 1b) and subgroups of antipsychotic drugs (Fig. 1c) for the different time periods.

## Discussion

This study analyzed pharmacotherapy of 1,044 psychiatric inpatients of German-speaking hospitals with the primary diagnosis of PTSD. Main result of our study was a high prescription rate of psychotropic medication (89.9%) with a majority of PTSD patients being prescribed more than one psychotropic drug (70.2%), mostly as a combination of an antidepressant and antipsychotic drug (45.2%). The psychotropic drugs with the highest prescription rates were quetiapine, lorazepam and mirtazapine—all of which are not approved for the treatment of PTSD in Germany, Austria and Switzerland. The analysis of time trends revealed an increased use of SGAs (mainly quetiapine) and a lower prescription rate of tranquilizing drugs over time.

High prescription rates have been observed in inpatients with other psychiatric disorders like BPD (90% [20]) and adjustment disorder to a similar degree (81.2% [21]). The high overall prescription rate of psychotropic drugs may seem surprising as psychopharmacotherapy is not recommended as first-line treatment for PTSD. However, inpatients with PTSD may exhibit high symptom burden and higher rates of therapy-resistance that leads to psychiatric admission and which may partially explain a more frequent use of psychotropics.

The mean number of psychotropics per patient with PTSD in this sample was 2.4 and therefore higher than in inpatients with adjustment disorder (2.1 per patient [21]) but lower than in inpatients with BPD (2.8 per patient [20]). The prescription rate in our sample was lower than in a large US veteran outpatient sample with PTSD (3.5 per patient [11]). However, the cohort described in this study differed greatly from our patient collective in regard to multiple factors like age, sex, comorbidity and symptom burden that may have had an impact on the prescription of medication. For instance, the veteran’s cohort consisted mainly of men (91.5% compared to 31.5% in our sample) with combat-induced trauma, that were older (average age 49.9 years) and suffered from a higher rate of psychiatric comorbidity than patients within our sample that may explain higher prescription rates (e.g., affective disorders: 58.9% vs. 27.8% in our sample; substance abuse: 33.7% vs. 13.6% [22]). Accordingly, we found that prescription rates of antidepressant drugs increased with age in our sample. In contrast, our analysis showed only slight differences between men and women regarding the use of specific psychotropics. As well, types of trauma may differ largely in the veteran population compared to our inpatient population.

The presence of an additional psychiatric diagnosis in our sample was associated with higher prescription rates of psychotropics, especially antipsychotic and “other antidepressant drugs”. However, this is surprising as only a minority of patients with PTSD in this sample suffered from comorbid affective (27.8%) and anxiety disorders (3.4%) that explain the use of antidepressant drugs. Furthermore, the low rates of comorbid schizophrenia and delusional disorders explain the extensive use of antipsychotic medication even less. Patients without comorbidities in our sample still showed a high prescription rate (86.0% psychotropics), with 83.8% of patients receiving psychotropic drugs that are not approved for the treatment of PTSD.

The prescription of psychotropics seems to be mainly symptom-driven with a considerable use of non-approved sedative drugs like quetiapine, lorazepam and mirtazapine that provide a fast-acting symptom relief. Indeed, hyperarousal is a core symptom of PTSD and sleep disorders are common due to insomnia and nightmares (e.g., 89% prevalence in a US veterans’ sample with PTSD [2]). A high symptom burden may even hinder the recommended psychotherapeutic treatment and therefore the experience-based prescription of sedative drugs may enable and support psychotherapy.

Models of PTSD suggest that psychotropic medication may address the PTSD related fear conditioning disturbances and stress-related synaptic alterations that result from a chronic stress pathology (for a detailed neurobiological model, see [23]). However, the exact mechanisms how psychotropic medication leads to a reduction of stress pathology and reduction of PTSD symptoms are not fully understood. Quetiapine had the highest overall prescription rate in our sample which further increased over time. Despite being not approved, SGAs are commonly used for patients with anxiety disorder and PTSD [24, 25] with an increasing trend [26]. Similarly, increasing rates of the prescription of quetiapine have been reported for patients with adjustment disorder [21] or BPD [20]. Quetiapine is usually well-tolerated without having an addictive potential as compared to benzodiazepines [21]. In addition to its sedative effect, quetiapine may prove potentially beneficial in the treatment of PTSD: in a placebo-controlled trial with 80 patients, Villarreal et al. [27] found quetiapine monotherapy given over a period of 12 weeks an efficacious treatment, particularly for symptoms like re-experiencing, hyperarousal and insomnia. However, the observed rate of remission was low [27, 28]. Other authors also observed improvement of insomnia [29] and nightmares [30] under treatment with quetiapine and describe anti-suicidal effects [31].

In contrast to the increasing prescription rate of quetiapine, the use of lorazepam showed a decline over time in our sample. Quetiapine may have been prescribed to control symptoms (e.g., arousal, suicidality) in lieu of the use of benzodiazepines, which are relatively contraindicated due to adverse drug reactions, addictive potential and worsened outcome of therapy (for a review, see [15]). However, the recommendation to avoid benzodiazepines may be confounded as more severely affected patients may both use benzodiazepines and profit less from therapy. The prescription rate of benzodiazepines within our patient sample were similar to US veterans’ samples (30.3% [11]), 14–23% [2]). Interestingly, these American samples showed a decline in benzodiazepine use over the last years and an increased prescription of zolpidem [2, 11], whereas we observed an increase of SGAs.

Mirtazapine was prescribed in high rates, however, there is limited knowledge about the efficacy of mirtazapine in the treatment of PTSD at present [32]. Several smaller scale studies suggest a potential effectiveness of mirtazapine as monotherapy [33–37]. The only placebo-controlled study with 29 patients found effectiveness on general anxiety symptoms but inconsistent results for different PTSD measurements over an 8 weeks observation period [38]. A 24-week placebo-controlled trial with 36 patients that compared the combination of sertraline and mirtazapine with sertraline and placebo showed no significant benefit of the combination drug therapy [39]. Regarding our observed prescription patterns, mirtazapine may have been chosen due to its sedative and antidepressant effects in order to treat insomnia and affective comorbidities of PTSD.

Prescription rates of approved drugs for PTSD, i.e., sertraline and paroxetine, were low within our sample and lower than among US veterans [11]. Whereas the prescription rate of sertraline was consistent over time, prescription rates of paroxetine even showed a decline, possibly in an effort to avoid adverse interaction effects of paroxetine by inhibition of CYP2D6 [40]. Despite recommendation of paroxetine and sertraline by guidelines (for instance German guidelines of AWMF [16]), clinicians may decide to prescribe other drugs due to their positive experience or due to potentially insufficient treatment response rates to the recommended drugs.

In general, a multitude of studies have shown that SSRIs are an effective treatment for patients suffering from PTSD with sertraline, paroxetine and fluoxetine being the most studied of this drug class in large caliber studies (for review [10, 12]). Further studies exist suggesting that other SSRIs, such as citalopram [41, 42] and escitalopram [43, 44] may also ameliorate symptoms of PTSD. However, for most of these drugs, randomized controlled studies are currently not available. Overall, SSRIs are postulated to affect serotonergic activity and modulate fear regulation in PTSD [45]. The effects of specific SSRIs may show some variability and the response to treatment may be related to trauma type, the duration and chronicity of PTSD [46]. Yet, Hoskins et al. [10] found insufficient evidence for using different SSRIs for different trauma types (combat vs. non-combat).

To summarize, our data shows a discrepancy between guidelines and psychiatric real-life prescription rates in patients with PTSD. There is a great demand for effective sedative agents such as mirtazapine and quetiapine for PTSD, suggesting that a great deal of suffering of PTSD inpatients may be due to hyperarousal and sleep disturbances. At the same time, there is a lack of high-quality evidence for many drugs that are prescribed under real-life conditions due to scarcity of randomized clinical trials. Further, the long-term outcome of these drugs in PTSD remains unclear. Future research should address the evaluation of currently prescribed drugs and the development of new and innovative substances by taking into account trauma type and comorbidities as this may affect treatment response [11, 47]. In addition, currently used psychotropics may valuably augment psychotherapeutic interventions. Therefore, the combination of psychotherapy and pharmacotherapy needs to be further studied as there is no clear evidence of an additional effect of combination therapy [8, 9, 45].

Strength of our study is the large sample of PTSD patients with representative comorbidity rates and a representative ratio of men and women for PTSD in Germany [5, 48]. Thus, our obtained data has a high ecological validity that represents psychopharmacological treatment in the natural clinical setting. However, several limitations have to be mentioned: our study analyzed prescription rates of psychiatric inpatients. This may result in higher prescription rates in comparison to outpatients, as inpatients may have a higher symptom load and symptom burden and may therefore require a more complex treatment. Furthermore, in case of medication changes with cross-tapering strategies prescription rates may be overestimated. In addition, group differences for sex, age and comorbidities may be overestimated as *p *values were not corrected for multiple comparisons. Multiple testing without correction can (inter alia) lead to false positive results, thus they have to be interpreted very carefully. The clinical severity of PTSD, trauma type and number of prior hospitalizations were not assessed in this sample. Furthermore, comorbidities may be underestimated as we do not have information whether comorbid diagnoses were assessed with standardized instruments in every contributing hospital. Finally, the available data did not include information on concurrent psychotherapy that may have interacted with prescription rates. However, the rate of psychotherapy can be assumed to be low as the main part of the participating psychiatric hospitals are not specialized in psychotherapeutic PTSD treatment.

To conclude, our study found that psychiatric inpatients with a primary diagnosis of PTSD show high rates of psychotropic drug prescription in clinical routine, particularly of sedative drugs like quetiapine and mirtazapine. This can be explained only to a minor extent by the presence of psychiatric comorbidities and points toward a symptom-oriented prescription. The high prescription rates in the absence of a comorbidity reveal a discrepancy of actually prescribed psychotropics and guideline recommendations that underlines the need for better pharmacological and psychological treatment strategies for PTSD in particular to address hyperarousal and insomnia. More medical trials, especially larger scale randomized placebo-controlled double blinded studies and naturalistic studies that include patients with specific comorbidities, are needed to further improve the treatment of PTSD.

## Data Availability

Data is available from the authors upon request.
